# Access to Assisted Reproductive Technologies in Chile

**DOI:** 10.5935/1518-0557.20140008

**Published:** 2014

**Authors:** Antonio Mackenna, Fernando Zegers-Hochschild

**Affiliations:** 1 Unidad de Medicina Reproductiva, Clinica Las Condes, Santiago, Chile; 2 Programa de Ética y Políticas Públicas en Reproducción Humana, Universidad Diego Portales, Santiago, Chile

**Keywords:** Postmortem, sperm retrieval, IVF

## Abstract

Assisted reproductive technology (ART) is present in almost all Latin American countries. However, it is accessible only to few couples, those who have the economic capacity to cover out of pocket funding. The number of initiated cycles per year in Chile covers less than 4% of the population who would theoretically need treatment. It corresponded to 609 cycles per 1.000.000 women between 18 and 45 years in 2009. This is in contrast with the reality of several European countries, where the numbers can be as high as 20,000 cycles per million women in reproductive age. Although availability of ART treatments is a reality in almost every country in the region, this inequality in the access is mainly due to socioeconomic reasons, which are discussed in this article.

Nowadays, the benefits of globalization are potentially enormous, as a result of a rapid transfer of technologies from developed to less developed countries, improving the quality of life. This happens with scientific and technological development related to assisted reproductive technologies (ART). However, the benefits of these treatments are far from reaching the majority of the Latin American population who need them. While technology is present in almost all countries in the region, it is accessible only to a few couples. This inequity in the access, present in Chile and other Latin American countries, is mainly due to socioeconomic conditions, generating an abysm between people who can and cannot afford ART. However, there are some psychosocial conditions involved in the low access to ART that must also be considered. Social organizations have emerged during the last decade and have been working together with the Chilean Fertility Society to change public health policies and attitudes in order to increase the access of people to modern reproductive technology.

## Chilean statistics and access to ART

**Figure 1:**
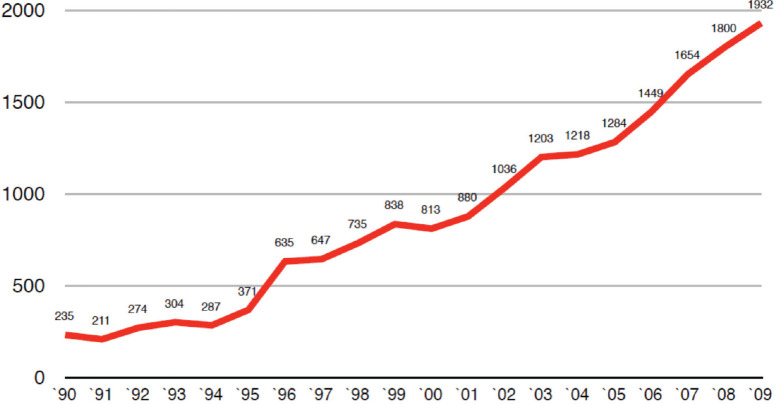
Number of assisted reproductive technology treatment cycles initiated per year in Chile from 1990 to 2009.

**Figure 2:**
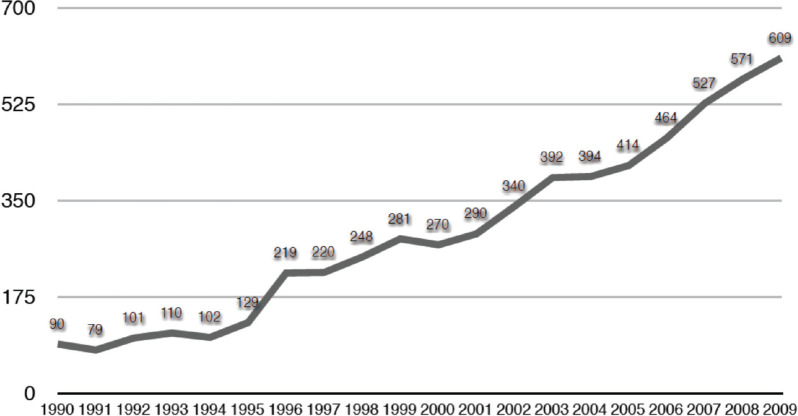
Access to assisted reproductive technology per year in Chile from 1990 to 2009, measured as the number of initiated cycles per 1.000.000 women within 18 and 45 years old.

Figure 1 shows the number of initiated cycles per year in Chile (IVF-ICSI-GIFT, oocyte donation and cryopreserved-thawed embryo transfer) and figure 2 the access to these kind of treatments, expressed as the number of initiated cycles per 1.000.000 women within 18 and 45 years old (reproductive age), with a maximum of 609 by 2009 (Schwarze *et al.*, 2009).

Although, the number of couples that can undergo these kind of treatments increases every year, the access of the population to them is still very low if compared with European countries, which have between 7.000 and 20.000 initiated cycles per 1.000.000 women in reproductive age per year (Nygren & Andersen, 2001).

On the other hand, assuming that 10% of women in their reproductive age are infertile and considering that 30% of them might require ART as part of their treatment, the number of treatment cycles performed per year in Chile in 2009 covered only 3.5% of the population who eventually needs treatment. This figure is similar than in other Latin American countries but, by far, below than the 30%, 40% and 95% observed in United Kingdom, France and Denmark, respectively (Zegers-Hochschild, 2002).

## Availability of ART

In 1990 only three centers had ART available in the capital city of Chile, and in 2009 nine centers were performing these treatments for Chilean couples across the country. The availability of the service is, indeed, one of the causes of the increase in the number of initiated cycles through the years. On the other hand, the fact that the government increased the number of financed treatment cycles per year from 50 to 250 for selected couples in the same period of time also contributed to the increase in the number of initiated cycles, although, less than 15% of the treatment cycles were subsidized by the government and the rest were performed in private hospitals or clinics that incorporate only those patients capable of affording around US$ 8.000 for each cycle. Therefore, in most of the cases access to ART means affordability.

## Socioeconomic conditions

There are two types of health insurance in Chile: a public system nominated *“FONASA”* (Fondo Nacional de Salud) and several private systems grouped under the name of *“ISAPRE”* (Instituciones de Salud Previsional). By law, 7% of taxable incomes are destined to cover health insurance. Individuals can decide whether to ascribe to the public system or to one of the private health institutions. The minimal costs of *“ISAPRES”* are, in general, higher than the public system and many times require complementary funds (*“out of pocket”* resources) for covering specific professional services. Overall, 60% of the population is part of the public system and 40% has a private insurance. The public system concentrates the segment of the population with the lowest incomes and with higher medical risks (population ≥ 65 years, irrespective of income). On the other hand, the private system attracts those segments with higher incomes and lower medical risks (younger population), (CASEN survey, 2011). This stratification of the population has a negative impact on the equity of the health system and on the access to high quality health care (WHO, 2011).

Table 1 examines the proportion of the Gross Domestic Product (GDP), which is a measure of the total goods and services produced by the economy over a period of time that is diverted into health expenditure between 2003 and 2010 in Chile, showing no significant changes throughout the years. It also provides some data on the proportion of public versus private as a percentage of the total health expenditure and the *“out of pocket”* health expenditure, corresponding to the percentage of the private health expenditure the patients have to pay directly without any reimburse (WHO, 2011). This is even worse in cases of ART, because, in the absence of a law to regulate ART and a null interest of *“FONASA”* and *“ISAPRES”* to cover high technology reproductive treatments, patients have to cover 100% of the costs of them. This is in contrast with many European countries, where the total amount of resources invested in health are 8% to 9% of the GDP and an average of 80% of the whole population is part of the public system, therefore having a more equal access to high technology health care and ART than in Chile (WHO, 2011).

**Table 1:** Health expenditure per year in Chile from 2003 to 2010.

Year	Total Health Expenditure (% GDP)	Public Health expenditure (*)	Private Health expenditure (*)	*“Out of pocket”* health expenditure (**)
2003	7.0	39.3	60.7	66.2
2004	6.6	40.1	59.9	67.1
2005	6.5	40.0	60.0	67.9
2006	6.2	42.0	58.0	68.8
2007	6.5	42.6	57.4	68.7
2008	7.1	43.5	56.5	69.9
2009	7.7	47.6	52.4	68.3
2010	7.4	47.2	52.8	69.1

(*) As % of total health expenditure.

(**) As % of private health expenditure. GDP: Gross Domestic Product.

The increase in the average annual income shown in table 2 might be a cause that contributes to the increase in the number on initiated ART cycles observed throughout the years in Chile, however, the vast majority of these are treatment cycles performed in private centers, where patients have to pay high costs for them. Actually, the average annual income alone doesnt mean that patients have access to high technology treatments. Table 2 also shows the corresponding Gini coefficient per year, which is a measure of statistical dispersion intended to represent the income distribution. A Gini coefficient of zero expresses perfect equality, where all values are the same (for example, where everyone has the same income) and a Gini coefficient of one expresses maximal inequality among values (for example where only one person has all the income), being Chile one of the countries with the highest coefficient in the world in contrast with the average Gini coefficient for European countries, which is 0.30 (Friedman & Hofman, 2013). Moreover, the official figures on income distribution in Chile show no significant progress in the last 22 years (Friedman & Hofman, 2013) and most of the couples needing modern reproductive technology to become parents cannot afford these treatments, although, the annual income increases every year and is expected to be US$ 20.000, one of the highest in Latin America, by 2014. This is in contrast to European countries, where most of the population has similar access to health services and modern technology, because high health expenditures and a public health structure covering the costs.

**Table 2:** Average annual income and Gini coefficient in Chile from 1997 to 2011.

Year	1997	2000	2003	2006	2009	2011
Annual income (US$)	8.557	9.229	10.379	12.737	14.700	17.400
Gini coefficient	0.57	0.58	0.56	0.53	0.53	0.52

US$: American Dollars.

## Psychosocial and ethical conditions

From the beginning of IVF in Chile (1984) an intense public resistance to ART was observed, basically coming from the moral position of the Catholic Church. However, during the last decade the concept of respect for the patient autonomy has changed public attitudes and the medical practice as well. Social organizations have emerged during the last years and are playing a key role in these changes.

A recent survey applied to a random representative sample of 1.500 people performed in Santiago, Chile, showed that 71.8% of respondents support ART to conceive, even by single women (70.4%) (Herrera *et al.*, 2013).

This issue also is contributing to the increase in the number of initiated cycles per year, although, the socioeconomic adverse conditions persist.
